# Novel Stenotic Microchannels to Study Thrombus Formation in Shear Gradients: Influence of Shear Forces and Human Platelet-Related Factors

**DOI:** 10.3390/ijms20122967

**Published:** 2019-06-18

**Authors:** Mathew Lui, Elizabeth E. Gardiner, Jane F. Arthur, Isaac Pinar, Woei Ming Lee, Kris Ryan, Josie Carberry, Robert K. Andrews

**Affiliations:** 1 Department of Mechanical and Aerospace Engineering, Monash University, 3800 Clayton, Australia; mathew.lui711@gmail.com (M.L.); isaac.pinar@monash.edu (I.P.); kris.ryan@monash.edu (K.R.); 2 Monash Institute of Medical Engineering, Monash University, 3800 Clayton, Australia; 3 Department of Cancer Biology and Therapeutics, John Curtin School of Medical Research, Australian National University, 2600 Canberra, Australia; elizabeth.gardiner@anu.edu.au; 4 Australian Centre for Blood Diseases, Monash University, 3004 Melbourne, Australia; jane.arthur@csl.com.au; 5 Research School of Electrical, Energy and Materials Engineering, The Australian National University, 2600 Canberra, Australia; steve.lee@anu.edu.au

**Keywords:** platelets, shear gradients, stenosis, thrombosis

## Abstract

Thrombus formation in hemostasis or thrombotic disease is initiated by the rapid adhesion, activation, and aggregation of circulating platelets in flowing blood. At arterial or pathological shear rates, for example due to vascular stenosis or circulatory support devices, platelets may be exposed to highly pulsatile blood flow, while even under constant flow platelets are exposed to pulsation due to thrombus growth or changes in vessel geometry. The aim of this study is to investigate platelet thrombus formation dynamics within flow conditions consisting of either constant or variable shear. Human platelets in anticoagulated whole blood were exposed ex vivo to collagen type I-coated microchannels subjected to constant shear in straight channels or variable shear gradients using different stenosis geometries (50%, 70%, and 90% by area). Base wall shears between 1800 and 6600 s^−1^, and peak wall shears of 3700 to 29,000 s^−1^ within stenoses were investigated, representing arterial-pathological shear conditions. Computational flow-field simulations and stenosis platelet thrombi total volume, average volume, and surface coverage were analysed. Interestingly, shear gradients dramatically changed platelet thrombi formation compared to constant base shear alone. Such shear gradients extended the range of shear at which thrombi were formed, that is, platelets became hyperthrombotic within shear gradients. Furthermore, individual healthy donors displayed quantifiable differences in extent/formation of thrombi within shear gradients, with implications for future development and testing of antiplatelet agents. In conclusion, here, we demonstrate a specific contribution of blood flow shear gradients to thrombus formation, and provide a novel platform for platelet functional testing under shear conditions.

## 1. Introduction

The rapid adhesion, activation, and aggregation of circulating platelets in flowing blood is crucial for initiating thrombus formation in hemostasis or thrombotic diseases. The capacity to form a thrombus depends on multiple parameters, including platelet-related factors (receptor expression and function, and reactivity towards various prothrombotic stimuli), vascular factors (exposure of prothrombotic surfaces by activation or disruption of endothelium), disease state comorbidities (atherosclerosis, inflammation, diabetes, or other prothrombotic conditions), and blood flow [1,2,3,4,5,6]. Currently available platelet functional testing based on platelet aggregometry is of limited value clinically, and even large-scale clinical trials have failed to show benefits in terms of antiplatelet therapy [7]. The potential value of testing pharmacological agents in microfluidic systems, in particular for screening drugs or combinations of drugs and determining effective doses and individual patient responses before or during treatment, has recently been evaluated using collagen-coated devices [8].

Platelet adhesion and thrombus formation in vivo is affected by the complex flow in the human circulatory system where arterial flows may be highly pulsatile and subject to temporal shear changes due to altered vessel geometries or occlusive thrombus. Platelets respond to changes in flow rate through mechanotransduction of forces that the flow generates [9,10,11]: a dominant aspect of these forces is the shear stress (force per unit area) due to velocity gradients that are inherently generated by the no-slip boundary condition at a vessel wall. Mechanotransduction relates directly to the reference frame (Lagrangian) of the platelet and is therefore independent of how the local shear environment is generated [12]. For example, equivalent shear gradients can be generated either by a temporally unsteady pulsatile flow in a straight vessel or by a temporally steady flow passing through an appropriately shaped stenosis. Determining the consequences of varying shear flows and shear gradients on platelet function and thrombus formation is critical for understanding targets/mechanisms of antiplatelet agents and development of new antithrombotic therapies.

To investigate platelet function within complex flow conditions experimentally, there are potentially two approaches to generating shear gradients. One method involves generating shear gradients by mimicking physiological pulsatile flows, for example, by using a variable pumping device. However, since platelets forming a thrombus in a temporally unsteady flow experience the full range of shears and shear gradients in the flow pulse, it is extremely difficult to identify how various components of the pulse are regulating platelet function. To overcome this problem, we utilized shear gradients generated by steady flow in spatially varying stenosis geometries, so that platelets adhering at a particular point have experienced the same known pulse of shear. Platelets adhering at different adhesion points experience different pulses of shear. As a flowing platelet moves through a stenosis, it experiences a period of increasing shear (positive shear gradient) followed by a period of decreasing shear (negative shear gradient). At the point where initial adhesion occurs, both the local shear magnitude and gradient as well as the shear histories are known and approximately temporally constant. As more platelets adhere and the wall surface shear is altered by growing thrombus geometries, values in the free-stream immediately prior to adhesion remain relatively unchanged.

In this study, human platelet function under flow conditions consisting of constant shear or variable shear gradients was investigated using collagen-coated microchannels, with variable wall shear rates and either constant shear in straight channels or shear gradients generated using varying stenosis geometries. Analysis of mature platelet thrombus formation by confocal imaging combined with computational flow-field simulations demonstrated specific outcomes of shear gradients on platelet function and thrombi formation, which improve understanding of pathophysiological factors regulating thrombus formation and have relevance for future development of antithrombotic agents.

## 2. Results

**Figure 1:**
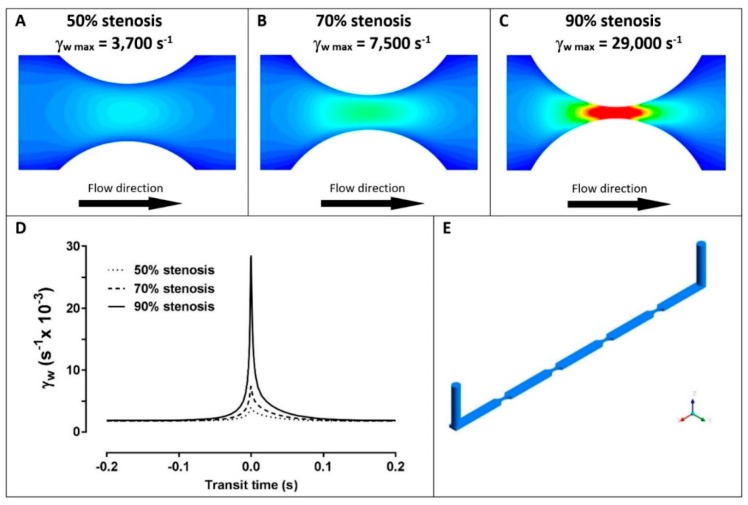
Shear stress in stenoses. The calculated shear fields and maximal wall shear rate (γ_w_, s^−1^) in microchannels with 50% stenosis (**A**), 70% stenosis (**B**)**,** and 90% stenosis (**C**). (**D**) The Lagrangian variation of wall shear (γ_w_, s^−1^) experienced by a platelet flowing along the stenosis centreline before and after the apex for 50% stenosis (dotted line), 70% stenosis (dashed line), and 90% stenosis (solid line). (**E**) Three-dimensional view of stenosis channel geometries showing the flow inlet and outlet.

Flow experiments were conducted in polydimethylsiloxane (PDMS) microchannels that were either straight channels with uniform rectangular cross-section (600 µm wide, 200 µm high) or stenosed channels with straight regions (600 µm wide, 200 µm high) separated by regions of stenosis with a diminished area (Figure 1). Each stenosis channel contained 4 consecutive stenoses of the same percentage area reduction (50%, 70%, or 90%), connected by regions of straight channel.

**Figure 2:**
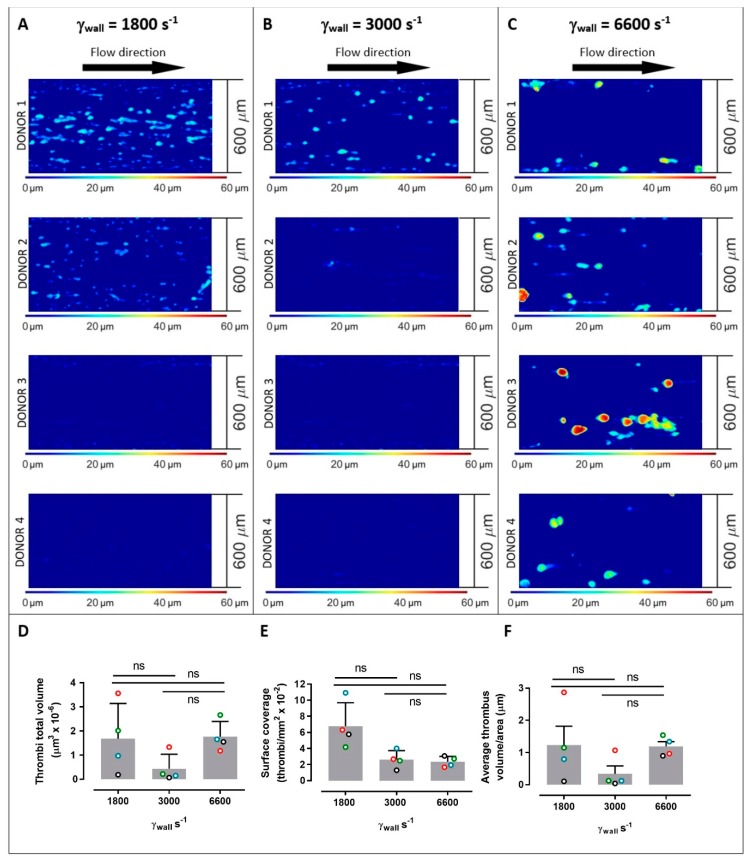
Platelet thrombus formation in straight channels. Images of thrombus formation (demonstrated by contours of thrombus height) all collected under the same conditions from anticoagulated blood from 4 separate donors within collagen-coated channels with constant wall shear rates (γ_w_) of 1800 s^−1^ (**A**), 3000 s^−1^ (**B**)**,** and 6600 s^−1^ (**C**) (donors 1–4 are shown from top to bottom panels at each shear rate). Measured thrombi total volume (**D**), surface coverage (**E**), and average thrombus volume/area (**F**) for 4 donors (open circles; red = donor 1, green = donor 2, blue = donor 3, black = donor 4) at 1800 s^−1^, 3000 s^−1^, and 6600 s^−1^, where bars represent mean ± standard deviation. Data in panels (**D**–**F**) were evaluated using one-way ANOVA with Tukey’s correction for multiple comparisons. ns = no significance. Larger numbers of smaller thrombi formed at 1800 s^−1^, while there were lower numbers of larger thrombi at 6600 s^−1^, and at the intermediate wall shear rate of 3000 s^−1^, thrombus formation was generally reduced suggesting distinct shear-dependent mechanisms regulating initial adhesion or growth of thrombi. Despite the differences in thrombus geometries, the total volumes of immobilised platelets at 1800 and 6600 s^−1^ were comparable (**A**).

**Figure 3:**
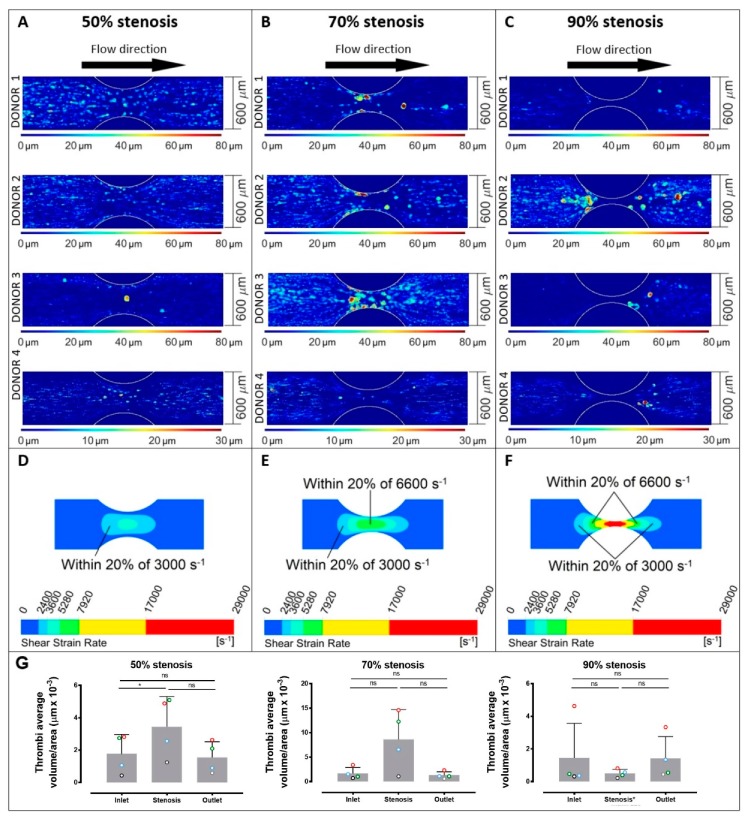
Platelet thrombus formation in stenosed channels. Images of thrombus formation (demonstrated by contours of thrombus height) all collected under the same conditions from anticoagulated blood from 4 separate donors within collagen-coated channels with 50% stenosis (**A**), 70% stenosis (**B**)**,** and 90% stenosis (**C**) (donors 1–4 are shown from top to bottom panels at each shear rate, and images represent overlays of four consecutive stenoses for each donor). Calculated wall shear patterns are shown for 50% stenosis (**D**), 70% stenosis (**E**)**,** and 90% stenosis (**F**), and thrombi average volume/area (**G**) are indicated for each donor (open circles; red = donor 1, green = donor 2, blue = donor 3, black = donor 4) at inlet regions, stenotic regions, and outlet regions for 50% stenosis, 70% stenosis, and 90% stenosis, where bars represent mean ± standard deviation. Data in panel (**G**) were evaluated using one-way ANOVA with Tukey’s correction for multiple comparisons. ns = no significance; * *p* ≤ 0.05. Pulsatile shear in stenotic channels compared with straight channels at varying shear (Figure 2) supports a specific role for shear gradients in initiating thrombus formation and regulating thrombus growth.

The three related parameters, thrombi total volume, surface area coverage, and average thrombus volume, were analysed in blood samples isolated from four different donors: data were obtained from thrombi formed at different wall shear rates (1800, 3000, or 6600 s^−1^) in straight channels (Figure 2) or with variable shear gradients using different stenosis geometries of 50%, 70%, or 90% by area (Figure 3). Note for direct comparison the thrombus height contours levels (Figure 2A–C and Figure 3A–C) are the same for all donors and all channel geometries. Computational flow-field simulations were also generated in the stenosed channels (Figure 3D–F). For subsequent analysis, imaging data from four separate stenoses in a given channel were overlaid (Figure 3A–C); there were no demonstrable differences between the patterns of thrombus formation in consecutive stenoses in an individual channel for a single donor.

In straight channels, the shear rates had a profound effect on the number and type of thrombi formed, with considerable variability between donors (Figure 2), about which several key points can be made. First, across all donors at 1800 s^−1^, larger numbers of smaller thrombi formed, while at 6600 s^−1^ there were lower numbers of larger thrombi. At these constant shear rates, the individual thrombi formed at 6600 s^−1^ had on average a 175% greater volume than thrombi formed at 1800 s^−1^, while there were 189% more individual thrombi at 1800 than at 6600 s^−1^ (Figure 2D). There were no significant differences in total volume, surface coverage, or thrombus volume/area when using input shear rate as the only variable in a 1-way ANOVA analysis. However, using 2-way ANOVA to simultaneously evaluate donor and shear rate variables, the input shear rate influenced thrombus surface area coverage (*p* = 0.0198) and accounted for 62% of the variance observed in this parameter. Donor-related factors did not significantly affect any of the parameters in Figure 2. Interestingly, at an intermediate wall shear rate of 3000 s^−1^, thrombus formation was generally reduced suggesting distinct shear-dependent mechanisms at elevated shear and greater dependence on growth of existing thrombi compared to initiation of new thrombi. That is, thrombus growth was more amenable at 6600 s^−1^ than platelet adhesion to the collagen surface, whereas at a lower shear rate (1800 s^−1^) platelet adhesion was relatively more achievable with greater limitations on thrombus growth. Therefore, this results in qualitative differences in types of thrombus formed at lower (1800 s^−1^) versus higher (6600 s^−1^) shear rates, and while the transition between these conditions appears to be donor-specific, a flex point commonly occurs at a shear of ~3000 s^−1^ where there appears clear to be reduced levels of thrombus formation. Despite the differences in the thrombus geometries, the total volumes of immobilised platelets at 1800 and 6600 s^−1^ were comparable.

In stenosed channels, increasing shear rates within 50%, 70%, and 90% stenoses also had a profound effect on the number and type of thrombi formed, and as for straight channels there were also marked differences between individual donors (Figure 3). In the stenosed channels, the variability in thrombus number and volume was related to the location within the stenosis, that is, either the inlet region, stenotic region, or outlet region (Figure 3G). In this case, the different percentages of stenosis present not only different values of wall shears but different gradients of shear (Figure 1). Despite differences between individual donors, the 50% stenosis showed an increased number of lower volume thrombi throughout the stenosis, the 70% stenosis showed a smaller number of larger thrombi within 300 µm of the stenosis, and the 90% stenosis also showed a smaller number of larger thrombi but predominantly 150 µm or more from the centre of the stenosis and with no stable adhesion within 150 µm (Figure 3). Using 2-way ANOVA analysis, the sampling position (inlet, stenosis, or outlet) and donor variables significantly affected the thrombus volume recorded (*p* = 0.0035 for position and *p* = 0.014 for donor) through the 50% stenosis. However at the 70% stenosis, only the sampling position significantly affected thrombus volume (*p* = 0.026). At the 90% stenosis, neither sampling position nor donor had any significant effect on thrombi volume. Notably, in the 50% stenosis, the differences in shear rates between the straight sections of the channels and the stenoses were relatively minor, with the peak shear in the stenosis at ~3000 s^−1^. In the 70% stenosis, larger stable thrombi tended to form towards the centre of the stenosis where the peak shear reached ~6600 s^−1^. In the 90% stenosis, larger thrombi formed mainly in inlet/outlet regions where the shear was 6600 s^−1^ or below, but not within the very high shear central stenosis region (peak shear up to 29,000 s^−1^) (Figure 3). However, although there was little or no stable adhesion within the centre of the 90% stenosis, video analysis showed a cycle of unstable contact adhesion and embolism.

While the three constant shear rates examined in the straight channels (Figure 2) are present in regions of the stenosed channels, a platelet moving through the stenosed channels experiences these levels in the context of shear gradients. The regions within the stenoses where shear amplitudes were within 20% of levels in the 3000 and 6600 s^−1^ straight channels are highlighted in Figure 3D–F. The shear gradients generated by the stenoses extended the range of shear rates at which thrombi were formed, that is, platelets became hyperthrombotic within shear gradients. In the 50% stenoses, where the surface area with shear rates in the order of 3000 s^−1^ was largest, pulsed flow passing through 3000 s^−1^ did not result in reduced thrombus formation (Figure 3A,E,G) as observed in steady flow at 3000 s^−1^ (Figure 2). In the 70% and 90% stenoses, the larger thrombi formed at lower shear locations (well below 6600 s^−1^) further indicated a role for shear gradients. The shear gradient effect appeared to be more pronounced in some donors and the threshold gradient at which it occurs may be donor-specific; donor 2 responded strongly to the 90% stenosis while donor 3 responded more to the 70% stenosis. Together, the differences in thrombus formation in both straight channels at varying shear and pulsatile shear in stenotic channels highlights a specific functional role for shear gradients in mediating thrombus formation, and suggest distinct shear-dependent pathways promoting either increased adhesion resulting in larger numbers of smaller thrombi or enhanced thrombus growth resulting in smaller numbers of larger thrombi.

## 3. Discussion

Improved understanding of how changes in shear stress and shear gradients regulate thrombus formation is critical for evaluation of hemostatic function and thrombotic risk in stenosed coronary vessels, as well as for identification of improved molecular targets and application of existing antiplatelet agents [1,2,3,4,5,6,7]. At arterial shear rates of 1800 s^−1^ or higher, key platelet-specific receptors glycoprotein (GP)Ibα of the GPIb-IX-V complex, and GPVI form a complex on the platelet surface and regulate binding of von Willebrand factor (VWF), collagen, and other prothrombotic/procoagulant ligands critical for thrombus formation under shear conditions [13,14,15,16]. Receptor expression and density specifically controls cell adhesion dynamics [17], and is critical for shear-induced thrombus formation [18].

Recent studies investigated how receptor expression and GPIbα/GPVI shedding contributes to donor-related differences and/or shear-dependent thrombus formation [13,19,20,21]. Exposure of human platelets to uniform shear stress ex vivo rapidly induces shedding of GPIbα and GPVI, while patients with cardiovascular disease, heart failure, and/or circulatory support devices (with acutely elevated fluid shear stress within these devices) show decreased platelet surface GPIbα/GPVI [1,20,21,22] and elevated shed soluble GPVI (sGPVI) in plasma, consistent with increased shedding [22,23]. There are now identified inter-subject variations that can exceed intra-subject variations up to 4-fold, in ex vivo thrombus formation that are due to multiple factors [24]. The latter could also include GPVI-linked genetic variations.

In summary, our new studies used computational flow-field simulations and measurement of thrombi total volume, average volume, and surface coverage in sequential stenotic micro-channels with geometries of 50%, 70%, and 90% by area to generate pulsatile shear and defined shear gradients. These methods can detect discrete differences in the initial adhesion and thrombus formation by human platelets that are not only varied between different donors, but are dramatically distinct from the thrombi formed with the same donor’s platelets in straight channels at constant wall shear rates of 1800, 3000, or 6600 s^−1^. Together, these findings provide a more patho-physiologically relevant analytical approach for future testing of antiplatelet targets or agents under pulsatile shear.

## 4. Materials and Methods

### 4.1. Blood Collection and Analysis

Whole blood was collected in accordance with the Declaration of Helsinki after informed written consent from 4 healthy volunteers, free of use of antiplatelet medication in the prior 10 days, using a 19-gauge winged infusion kit as approved by the Monash University Standing Committee for Research in Humans (CF07/0141 – 2007000025, issued 14.01.16). The blood was collected into hirudin (800 U/mL) anticoagulant and platelet membranes stained with a fluorescent dye (DiOC_6_) to enable confocal imaging [12].

### 4.2. Fabrication of the Stenosed Microchannels

The PDMS microchannels were fabricated using a standard PDMS soft lithography method where PDMS was casted onto a master containing the negatives of the channels. The master was created using standard photolithography techniques where two layers of 100 µm-thick SU-2075 (MicroChem, Westborough, MA, USA) photoresist were initially spin-coated onto a clean silicon wafer. After the spin-coating first and second photoresist layer, the layers were baked for 40 and 90 min, respectively, at 95 °C. The patterns of the channels were exposed onto the photoresist using 400 mJ/cm^2^ of ultraviolet light. After the exposure, the photoresist was further baked for 90 min at 95 °C followed by development in SU-8 developer (MicroChem) to remove unexposed photoresist. The silicon wafer was then silanized (20 µL of Trichloro (1*H*,1*H*,2*H*,2*H*-perfluorooctyl) silane (Sigma-Aldrich, Castle Hill, NSW, Australia)) before casting with Sylgard-184 PDMS (Dow Corning, Midland, MI, USA) with a curing agent-to-base weight ratio of 1:10. The cast was cured at 65 °C for 5 h, and inlets and outlets of the channels were cut using a 0.75 mm hole puncher. The channels were sealed using #1.5H coverslips (Menzel-Gläser, Braunscheig, Germany) bonded using air plasma at 300 mTorr for 45 s.

### 4.3. Calculations of the Stenosis Percentages

Periodic stenoses in the channel cross-sectional area were created by two parallel channel walls with circular segment profiles where each had a radius of 0.5 mm. The stenosis percentage (${\%}_{sten}$) was defined by

$${\%}_{sten}=\frac{A_{non-sten}-A_{sten}}{A_{non-sten}}\times 100\%,$$

where $A_{sten}$ is the minimum channel cross-sectional area within the stenosis and $A_{non-sten}$ is the channel cross-sectional area of the non-stenosed regions of the channel.

### 4.4. Platelet Thrombus Formation under Flow

Microchannels were cleaned with 80% (*v*/*v*) ethanol followed by Tris-saline (TS) buffer (0.01 M Tris-HCl, 0.15 M NaCl, pH 7.4) and then coated with bovine type 1 collagen (100 µg/mL) as previously described [11]. Blood was perfused through the channels for 3 min using a syringe pump (Harvard Apparatus PHD2000, Holliston, MA, USA) in refill mode, followed by buffer flow to flush red blood cells. The thrombus geometries were then fixed using 1% (*w*/*v*) paraformaldehyde solution, and the microchannel was sealed prior to scanning. The pulsed studies were conducted with a base wall shear rate of 1800 s^−1^ from which platelets were subjected to elevated shear pulses using three different stenosis geometries, 50%, 70%, and 90% by area. The flow fields within channels were simulated using the ANSYS CFX solver, revealing the spatial variation of shear within each stenotic region (Figure 1). The temporal variation of the shear experienced by a platelet flowing along the centreline of the channel just above the wall was also calculated (Figure 1D), with positive shear gradients upstream of the apex and negative shear gradients downstream. Platelet adhesion in the stenosis geometries with pulsatile shear was also compared to flow adhesion assays in straight channels at different shear rates (γ_wall_) of 1800, 3000 and 6600 s^−1^, where these values were selected to correspond to key shear rates within the stenosis channels.

### 4.5. Analysis of Thrombus Formation

Sections of the channels were imaged using confocal fluorescence microscopy (Nikon A1r, 512 × 512 pixel image resolution; Melville, NY, USA) to acquire overlapping z-stacks with Δz of 0.7 µm. The z-stacks captured all thrombi adhered to the bottom surface of the microchannels within the x-y scan area. The thrombus geometries were isolated using a density-based segmentation algorithm [25] and the floor of the channel identified, allowing for correction for channel tilt during scanning.

### 4.6. Shear Force Equations and Units

The force generated by velocity gradients was given (in two-dimensions for simplicity) by:

$$F=\mu \frac{du}{dy}A$$

where *µ* is the dynamic viscosity (~3.2 × 10^–3^ Pa·s for whole blood), $\frac{du}{dy}$ is a velocity gradient (the rate at which the velocity changes in the direction normal to which it is travelling), and *A* is the area over which the force is acting. The velocity gradient is also known as the shear rate (units of inverse seconds) and is directly proportional to the shear-induced force exerted by the flow on a platelet. The shear rate varies across a blood vessel but is often characterised by the shear rate at the vessel wall or wall shear γ_wall_ = ${\frac{du}{dy}]}_{y=0}$, where the shear at the wall is typically maximised and also most relevant to platelet adhesion. The shear rates calculated within the stenosed microchannels were three-dimensional and derived from the three-dimensional strain rate tensor. For completion, the shear rate in the three-dimensional space was given by:

$$\dot{\gamma }=\sqrt{2\left[{\left(\frac{\partial U_{X}}{\partial X}\right)}^{2}+{\left(\frac{\partial U_{Y}}{\partial Y}\right)}^{2}+{\left(\frac{\partial U_{Z}}{\partial Z}\right)}^{2}\right]+\left[{\left(\frac{\partial U_{X}}{\partial Y}+\frac{\partial U_{Y}}{\partial X}\right)}^{2}+{\left(\frac{\partial U_{X}}{\partial Z}+\frac{\partial U_{Z}}{\partial X}\right)}^{2}+{\left(\frac{\partial U_{Y}}{\partial Z}+\frac{\partial U_{Z}}{\partial Y}\right)}^{2}\right]},$$

where $\dot{\gamma }$ is the shear rate, X, Y, and Z are the orthogonal directions in the 3-dimensional space, and U is the velocity; the subscripts denote the directional components of the velocity.

## 5. Conclusions

These studies demonstrate key differences in thrombus formation in straight channels at varying shear and at pulsatile shear in stenotic channels, and support a specific functional role for shear gradients in mediating initiation and growth of thrombi involving human platelets, with contributions also from platelet-specific parameters. These data have implications for understanding mechanisms for thrombus formation under altered flow conditions in disease and clear implications for targeting and monitoring antiplatelet therapies in future.
